# RoboDiff: combining a sample changer and goniometer for highly automated macromolecular crystallography experiments

**DOI:** 10.1107/S205979831601158X

**Published:** 2016-07-27

**Authors:** Didier Nurizzo, Matthew W. Bowler, Hugo Caserotto, Fabien Dobias, Thierry Giraud, John Surr, Nicolas Guichard, Gergely Papp, Matias Guijarro, Christoph Mueller-Dieckmann, David Flot, Sean McSweeney, Florent Cipriani, Pascal Theveneau, Gordon A. Leonard

**Affiliations:** a European Synchrotron Radiation Facility, 71 Avenue des Martyrs, CS 40220, F-38043 Grenoble, France; b European Molecular Biology Laboratory, Grenoble Outstation, 71 Avenue des Martyrs, CS 90181, F-38042 Grenoble, France; c Unit for Virus Host Cell Interactions, Université Grenoble Alpes–EMBL–CNRS, 71 Avenue des Martyrs, CS 90181, F-38042 Grenoble, France

**Keywords:** RoboDiff, automation, robotics, high throughput, goniometers

## Abstract

Automation of the mounting of cryocooled samples is now a feature of the majority of beamlines dedicated to macromolecular crystallography (MX). Robotic sample changers have been developed over many years, with the latest designs increasing capacity, reliability and speed. Here, the development of a new sample changer deployed at the ESRF beamline MASSIF-1 (ID30A-1), based on an industrial six-axis robot, is described. The device, named RoboDiff, includes a high-capacity dewar, acts as both a sample changer and a high-accuracy goniometer, and has been designed for completely unattended sample mounting and diffraction data collection. This aim has been achieved using a high level of diagnostics at all steps of the process from mounting and characterization to data collection. The RoboDiff has been in service on the fully automated endstation MASSIF-1 at the ESRF since September 2014 and, at the time of writing, has processed more than 20 000 samples completely automatically.

## Abstract 2

An industrial six-axis robot has been combined with a high-accuracy air-bearing rotation axis to create a single device with the capabilities of both transferring cryocooled protein crystals from a sample-containing dewar and collecting complete X-ray diffraction data sets.

## 1. Introduction   

The automation of macromolecular crystallography (MX) experiments on synchrotron beamlines is aimed at increasing throughput and at simplifying data-collection and beam-alignment protocols in what can be a complicated and stressful environment for novice users. It is the result of the blending of a wide variety of different software developments including graphical user interfaces (GUIs) for beamline control (Gabadinho *et al.*, 2010 ▸; McPhillips *et al.*, 2002 ▸; Stepanov *et al.*, 2011 ▸; Fodje *et al.*, 2012 ▸), online data analysis (Dauter, 1999 ▸; Holton & Alber, 2004 ▸; Incardona *et al.*, 2009 ▸; Leslie *et al.*, 2002 ▸; Sauter *et al.*, 2004 ▸) and laboratory information-management systems (LIMS; Delagenière *et al.*, 2011 ▸; Fodje *et al.*, 2012 ▸) with hardware advancements such as the standardization of sample holders and the development of robotic sample changers (Cipriani *et al.*, 2006 ▸; Cohen *et al.*, 2002 ▸; Jacquamet *et al.*, 2009 ▸; Ohana *et al.*, 2004 ▸; Pohl *et al.*, 2004 ▸) that automate the transfer of cryocooled protein crystals to a goniometer prior to data collection. The automation protocols available at nearly all synchrotron-based MX beamlines now routinely allow the collection and analysis of diffraction data from several hundreds of crystals in a typical experimental session (Beteva *et al.*, 2006 ▸; Bowler *et al.*, 2010 ▸; Elsliger *et al.*, 2010 ▸; Ferrer *et al.*, 2013 ▸; Heinemann *et al.*, 2003 ▸; Holton & Alber, 2004 ▸; Malbet-Monaco *et al.*, 2013 ▸; Monaco *et al.*, 2013 ▸; Nurizzo *et al.*, 2006 ▸; Okazaki *et al.*, 2008 ▸) and, in principle, allow their completely unattended operation. However, until recently the small capacity of the sample-containing dewar associated with robotic sample changers and the need for manual interventions to recover from sample-changer errors has made this possibility unworkable. The standard mode of operation of MX beamlines is thus that data collection is still carried out by users, either present at the beamline or remotely.

To date, two main approaches have been followed to construct sample changers (for an overview, see Ferrer *et al.*, 2013 ▸). The first approach is based on a dewar with motorized rotation and translation axes for sample positioning and sample transfer, for example SC3 (Cipriani *et al.*, 2006 ▸). The second approach transfers samples by picking them directly from a standard dewar and placing them onto a goniometer using a multiple-axis robot, for example CATS (Jacquamet *et al.*, 2009 ▸) and ACTOR (Rigaku). In both of these cases the sample changer is uncoupled from the goniometer. A third approach comprises the use of an anthropomorphic robot arm to both transfer the samples and carry out data collection. This approach was pioneered by the French beamline for the Investigation of Proteins (FIP; BM30B) at the ESRF, Grenoble with the development of the G-Rob (Jacquamet *et al.*, 2004 ▸; Nat-X-Ray, Grenoble, France). Here, a six-axis robot arm coupled with a magnet mounted on a mechanical gonio­meter head which is in turn mounted at the extremity of the arm allowed the combination of sample-changer and gonio­meter functionalities. While innovative, the G-Rob suffered from several disadvantages, the most important being the dynamic correction required to reduce the sphere of confusion (SOC) during a complete rotation of the goniometer. This prevented the use of pixel-array detectors in continuous readout mode and significantly reduces potential beamline throughput (Hülsen *et al.*, 2006 ▸; Ferrer *et al.*, 2013 ▸). Additionally, a complete absence of diagnostics (such as detection of the presence or absence of a sample) made the autonomous operation of the G-Rob problematic.

Taking this knowledge into account, the ESRF launched a project to build a sample changer coupled to a built-in goniometer with the specifications required to be able to fully exploit high-brilliance undulator beamlines equipped with pixel-array detectors. The resulting RoboDiff device includes a high-capacity dewar (HCD) that is able to accommodate 240 SPINE standard sample holders (eight cells of three pucks each, giving a total of 240 samples), a six-axis robot for sample transfer and a high-accuracy air-bearing rotation axis acting as the goniometer. A dedicated effort has been made to integrate a high level of diagnostic information at all stages of the operation of the device in order to trace errors and, importantly, for the device to recover from these conditions. Careful attention has also been paid to sample identification, which is essential for automated user-free data collection. The RoboDiff forms the core of the services offered on the ESRF beamline MASSIF-1 (Bowler *et al.*, 2015 ▸, 2016 ▸; Svensson *et al.*, 2015 ▸) and, thus far, has been used to mount, characterize and collect diffraction data from over 20 000 samples since September 2014.

## 2. Experimental details   

The RoboDiff consists of an HCD coupled to a six-axis robotic arm mounted with a stack comprising an air-bearing rotation axis, centring tables and an electromagnet. The device can be divided into two highly correlated entities: the sample changer that performs the transfer of samples from the HCD to the beam position, and the goniometer that performs the data collection. The main advantage of this approach is that many of the parts are used by both components. For example, the same electromagnet is used to maintain the sample pin in position during data collection and for picking the sample from the HCD. By reducing the complexity of the system and integrating all functions in a single device, error handling and troubleshooting are efficiently treated at a low level, with a net gain in reliability.

### 2.1. The RoboDiff as a sample changer   

**Figure 1:**
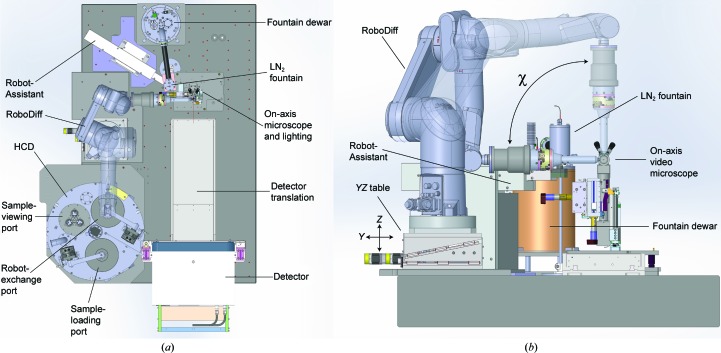
Schematic of the RoboDiff, HCD and experiment environment on MASSIF-1. (*a*) Top view of the table. The RoboDiff is shown in parked (transparent) and data-collection orientations. (*b*) The RoboDiff viewed from the detector. Two values of χ are shown (with 90° transparent). Vials are removed at the χ = 90° position and data collection can be performed at any angle between 0 and 90°. Vertical and horizontal translations (*X*/*Y*) are performed using the table mounted below the robot.

**Figure 2:**
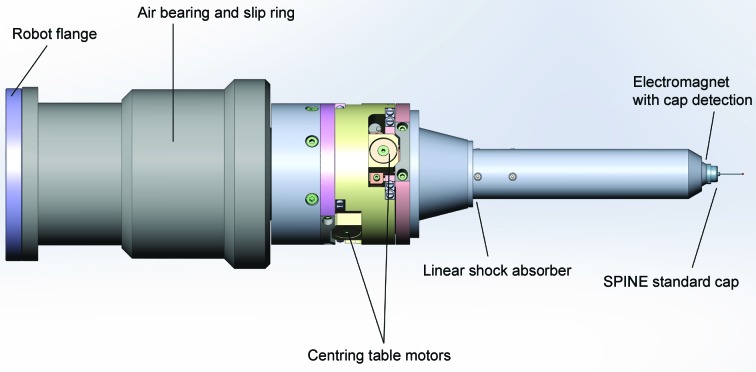
Schematic of the RoboDiff head. The components of the high-accuracy spindle are shown. The assembly is mounted on the sixth axis of the robot and is composed of the air bearing, centring table, shock absorber and electromagnet. The device has a sphere of confusion of 1.4 µm.

The RoboDiff is based on a Stäubli TX60L (Stäubli Faverges SCA, Faverges, France) with six degrees of freedom (Fig. 1 ▸). The TX60L was chosen for its higher radius of work compared with the TX60, which is also available (920 *versus* 670 mm). The nominal weight capacity of the TX60L is 2 kg, approximately the weight of the high-precision air-bearing rotation axis, centring tables and electromagnet mounted on the sixth axis of the robot (Fig. 2 ▸, §[Sec sec2.2]2.2). The robot is controlled by a CS8C controller (Stäubli) with management of input/output (I/O) performed *via* an ethernet controller (Modbus/TCP Ethernet controller 5102-3997) specifically developed for the CS8C controller (WAGO Contact SAS, Roissy, France). This configuration allows the control of all I/O devices linked to the RoboDiff, such as opening and closing of the dewar pneumatic ports (§[Sec sec2.3]2.3), driving the pneumatic jacks of the Robot-Assistant and controlling image-recognition cameras; it leads to highly reliable cross-checking and synchronization of RoboDiff movements.

Sample mounting proceeds by moving the robotic arm to the position of a SPINE sample holder contained in the HCD. The electromagnet in the head is then used to pick the entire sample holder (*i.e.* both pin and vial) from the puck. Maintaining the sample holder in the vertical position (χ = 90°; Fig. 1 ▸), the robot then moves to the beam position, where removal of the vial is performed by a Robot-Assistant composed of a vial support and two pneumatic translations, allowing independent in/out and up/down movements. The vial support, which is kept in a stream of gaseous nitrogen to prevent ice formation, uses an electromagnet to attract the metal base of the vial and an optical fibre to detect the presence of a vial (*i.e.* that it has been correctly removed). Once the vial has been removed successfully, the Robot-Assistant moves itself, and the vial that it contains, to a park position 20 cm away from the beam position. This is located beneath the liquid-nitrogen (LN_2_) injector of the LN_2_ fountain. This fountain is composed of a small dewar (1.5 l) and a pump that fills an injector with a 5 ml volume, approximately twice the volume of a SPINE standard vial. However, during data-collection procedures the vial remains at room temperature. The injection of liquid nitrogen is activated only when the unload procedure starts, and the vial is filled with LN_2_ during the time it takes for the robot to move from the horizontal to the vertical position. With the sample now free of its vial and maintained in a stream of gaseous nitrogen (100 K) provided by an Oxford Cryosystems 700 Series Cryostream, the robotic arm is then moved to χ = 0° (horizontal) and the sample is ready for alignment in the X-ray beam.

For sample unloading, the above sequence is performed in reverse order: the robot arm moves to χ = 90°, the Robot-Assistant returns from its park position, the sample and vial are reunited and are then returned to the HCD. However, immediately before the Robot-Assistant returns from its park position, in order to ensure that sample integrity is maintained during the unloading procedure, an electro-valve, controlled by the robot, is opened to allow the refill of the vial by the LN_2_ fountain. The LN_2_ levels in both components of the fountain are controlled using PT100 temperature probes connected to dedicated WAGO modules which also control the I/O modules of the valves. In order to reduce initial problems owing to the variation of pressure in the main ESRF LN_2_ feeding network, an automatically filled 120 l dewar (XRP120; Cryo Diffusion, Léry, France) supplying both the LN_2_ fountain and the cryostream has been installed. This buffer dewar is pressurized to 100 kPa and its installation has proved to be a major step forward in the stability and reliability of the system.

The alignment of the robot with respect to the HCD is crucial in order to correctly position it for sample transfer. To facilitate this alignment over the 24 pucks, a dedicated tool has been designed that is attached to the tip of the robot. This device includes a limit switch and an automatic procedure has been programmed to define the centre of each puck inside the dewar. The routine defines the centre of each puck (in the HCD the 24 pucks are divided into eight cells of three pucks each) to a precision of ±50 µm.

### 2.2. The high-accuracy goniometer   

While the TX60L, with a repeatability of positioning of ±30 µm (manufacturer’s documentation; Stäubli Faverges SCA, Faverges, France), is excellent for the pick-and-place movements required for the mounting and unmounting of protein crystals in SPINE standard sample holders, a much higher level of precision is required for the manipulation of, and data collection from, small crystals in a ∼50 µm diameter X-ray beam (the default beam size of MASSIF-1). Motions of the robotic arm are thus only used during the loading and unloading procedure.

Once the robotic arm is at χ = 0°, the sample is optically aligned to the X-ray beam position using the robot translations. For subsequent alignments, the *Y*/*Z* translation (Axmo, Brétigny-sur-Orge, France) of the table installed beneath the robot is used (Fig. 1 ▸ *b*). This translation table has a range of ±1.4 mm for both vertical and horizontal movements, which are encoded to 120 and 20 counts µm^−1^, respectively. Owing to the repeatability error of the robot when bringing the robot arm to χ = 0°, the centre of rotation of the air-bearing rotation axis with respect to the beam position must be aligned after each loading procedure. This is performed during subsequent X-ray crystal-centring protocols, the sample translations for which are also carried out using the motors of this trans­lation table (Svensson *et al.*, 2015 ▸).

Standard data collection by the rotation method is performed using the high-precision air-bearing rotation axis (Nelson Air Corp., Milford, New Hampshire, USA) mounted on the sixth axis of the robot (Figs. 1 ▸ and 2 ▸). This is fitted with an 18-wire slip ring allowing infinite rotation of the ω axis. Its brushless motor is controlled independently, emulating a stepper-motor device, and is connected to an ESRF ‘Intelligent Controller for Positioning Applications’ (ICEPAP) motor controller that treats the axis like an encoded stepper motor (resolution 13 152 steps deg^−1^). The encoder signal is split, going to both the air-bearing controller (internal closed-loop system, control of movements *etc.*) and to a Multipurpose Unit for Synchronization, Sequencing and Triggering (MUSST) synchronization card (see §[Sec sec3]3). The alignment of a crystal with the rotation axis is performed using a centring table with two axes 90° apart (ARINAX, Moirans, France; Fig. 2 ▸) mounted on the air bearing. The range of the two axes is ±2 mm in both directions and the table is equivalent to that mounted on MD2 microdiffractometers (Perrakis, Cipriani *et al.*, 1999 ▸). An electromagnet and a pin detection system based on a photo-interrupter (SG-2BC, Kyoto, Japan) have been mounted on the centring table (Fig. 2 ▸). A small electronic interface has been developed to set the trigger level for the presence or absence of a pin on the electromagnet. In order to avoid mechanical shocks to the centring table during sample loading and unloading, the magnet nozzle contains a shock-absorber shaft fitted with a spring (Fig. 2 ▸).

The sphere of confusion (SOC) of the air bearing alone has been measured as 250 nm with the rotation axis in a horizontal orientation. By stacking the centring table and electromagnet nozzle onto the air bearing, this SOC is increased to ∼1.4 µm (robot arm at χ = 0°), a value comparable to that of the MD2 (Perrakis, Cipriani *et al.*, 1999 ▸). The performance of the device has been demonstrated from data collection on a wide variety of crystals from users, including one with dimensions of 28 × 57 × 13 µm, which is indicative of the accuracy of the device (Bowler *et al.*, 2016 ▸).

Extra functionality has been added to the RoboDiff by locating an X-ray scintillator screen fitted on a SPINE standard pin next to the beam position that can be automatically placed at the beam position by the robot. This option allows visualization of the beam at the sample position without manual intervention.

### 2.3. The high-capacity dewar   

**Figure 3:**
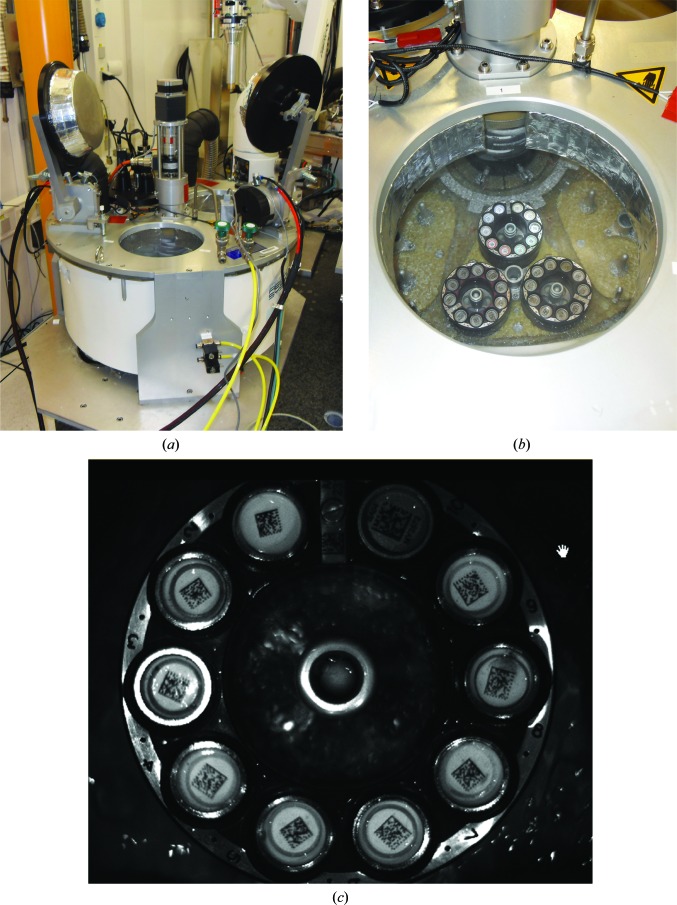
The HCD. (*a*) The HCD is shown with the sample-loading port (foreground) and robot-transfer ports open. The sample-loading port allows the loading of new pucks without interupting robotic operations. The rotation motor is located at the centre outside the dewar in order to reduce mechanical blockage owing to icing. (*b*) Inside the HCD. One of the eight cells each with three positions for SPINE pucks is shown. The LN_2_ level is maintained at ±5 mm from the top of the puck. (*c*) Image of a puck from the dewar camera. The image allows the barcodes to be read and the presence or absence of pins, pucks and vials to be determined.

The RoboDiff mounts and unmounts samples to and from an HCD made from polyurethane foam and which has a maximum capacity of 240 SPINE standard sample holders in 24 pucks (Figs. 1 ▸ *a* and 3 ▸). The HCD consumes around 1.5 l h^−1^ of LN_2_ and its interior is maintained at slightly above atmos­pheric pressure. Ambient humidity is therefore kept outside the HCD, dramatically reducing ice formation. The LN_2_ level in the HCD is maintained around the top of the pucks. The vials in which the samples are contained are thus always full of LN_2_ when in the HCD (Fig. 3 ▸ *b*). Two independent access ports allow the filling of the HCD with samples without interfering with sample transfer, while a third port is dedicated to the bar-code reader cameras necessary for the full tracking of samples during an experiment (Figs. 1 ▸ *a* and 3 ▸ *a*, see below). In the HCD, the 24 pucks are divided into eight cells of three pucks each (Fig. 3 ▸ *b*) bolted to a rotating plate. The motor that drives the rotation of the cells is mounted outside the HCD in order to avoid the difficulties associated with movements under LN_2_ (Fig. 3 ▸ *a*). This rotation-axis motor is mechanically mounted with an absolute encoder (resolution 8192 steps per turn) in order to track the exact position of a cell within the HCD (Fig. 3 ▸ *a*).

Both SPINE standard sample holders and pucks have an individual two-dimensional data-matrix code for identification. The HCD includes a set of three cameras (Keyence, Courbevoie, France) located on one port in order to rapidly identify samples in the HCD (Figs. 1 ▸ *a* and 3 ▸ *c*). The system determines the data-matrix code on each sample and puck and also checks for the presence or absence of vials, pins and pucks in order to prevent crashes. The three cameras [CV200M (1600 pixels × 1200 pixels) with CA-LH16 lenses (focal point 16 mm)] are controlled with a CVX-150FP controller which also stores and runs the detection programs (defining bar-code reading, shape-recognition and image-recognition algorithms). An ethernet socket is used to swap between the different detection options and to retrieve the results from diagnostic runs and barcode reading. The lighting system, a crucial part of the detection, is composed of rings of three LEDs (HLMP-EH08-WZ000; Avago Technologies, Singapore) for each puck, located at the entrance of the port. They are mounted on a rigid support and bolted on the sample-viewing port (Fig. 1 ▸).

**Figure 4:**
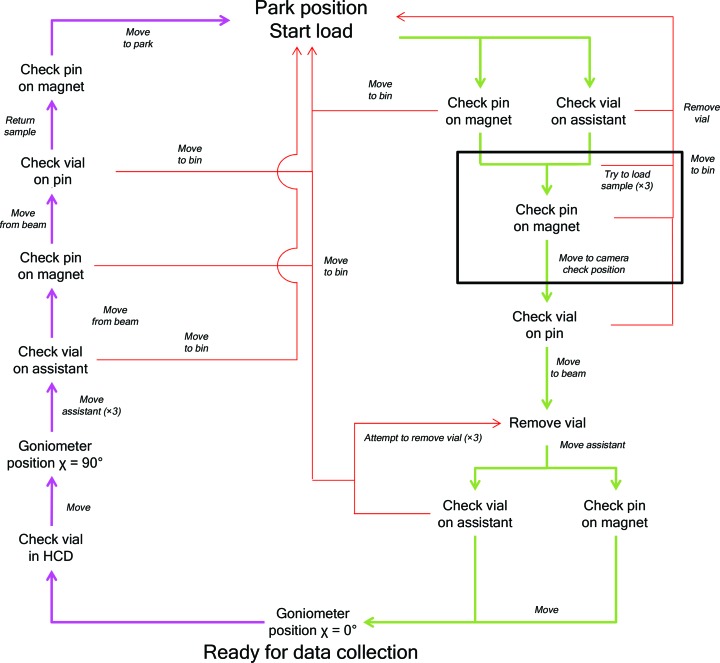
General workflow for sample transfer. The park position is the initial position and the goniometer position χ = 0° is the standard horizontal geometry for data collection. Green and red arrows indicate the unencumbered path taken to load and unload a sample, respectively. Red arrows indicate the actions taken in case of errors and the route taken to continue with the current sample or move to the next sample. The black box shows steps that occur in the HCD.

In addition to the detection of samples within the HCD, an optical detection of vial presence on the sample pin is performed as samples leave and enter the HCD (Fig. 4 ▸). This is performed using a Keyence IV Series 500MA camera (Keyence, Courbvoie, France). The camera is triggered externally and the results are retrieved by the robot controller.

### 2.4. Software integration   

**Figure 5:**
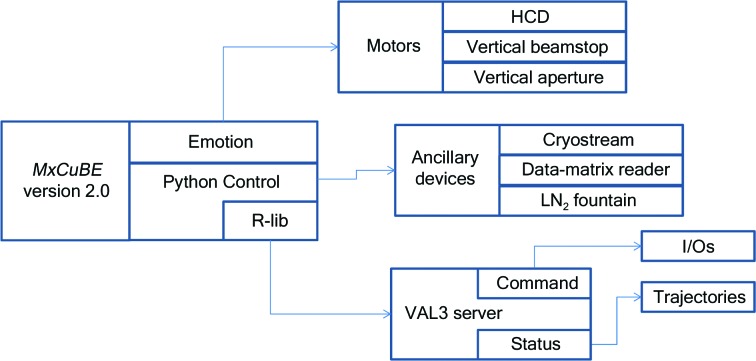
Software architecture and control of the RoboDiff. The control is intrinsically linked to *MXCuBE* 2.0, which plays a central role in the automation of MASSIF-1.

The functions of the RoboDiff are programmed within the CS8C controller. Routines are written in VAL3 using the *Stäubli Robotics Suite* v.7.3 (Stäubli Faverges SCA, Faverges, France). Trajectories and logic to be applied during robot movements are defined as tasks that can be launched by the *StaubCom* (EMBLEM Technology Transfer GmbH, Heidelberg, Germany; http://software.embl-em.de) generic VAL3 socket server on the controller side. The *StaubCom* library communicates through an ethernet connection and uses two independent ports dedicated to command and update services. The command service is used to receive keep-alive messages from the client, to launch or abort tasks and to modify VAL3 global variables on the fly. The update service sends asynchronous notification messages to the client machine including the status of the robot, input/output states and log or exception messages (Fig. 5 ▸). A Python library (R-lib) acts as the client of the *StaubCom* server, linking it to the beamline-control GUI *MXCuBE* (Gabadinho *et al.*, 2010 ▸).

All VAL3 routines are made available to user as Python functions (Python Control). Other ancillary devices and motors in the sample environment such as the dewar, actuators or additional motors are integrated in the standard beamline-control software. The library provides high-level Python objects both for convenience and for integration purposes. The main RoboDiff functions are wrapped into distinct objects depending on their role. Sample identification (data-matrix reading) and handling functions (mounting and unmounting) can be called as part of a generic Sample Changer object in *MXCuBE* 2.0. Oscillation and helical scans are provided by a Diffractometer object. RoboDiff axes are treated as any other motorized axis on the beamline. As a result, it is possible to extend RoboDiff functionality in Python.

### 2.5. Sample-transfer and data-collection procedures   

Mounting and unmounting cycles follow a logical flow where decisions are made at various diagnostic points (Fig. 4 ▸ and Table 1 ▸). Prior to any RoboDiff movements, the desired HCD cell is moved to the loading position and the Pilatus3 2M detector, beamstop and apertures of the beamline (for details, see Bowler *et al.*, 2015 ▸) are moved to safe positions. These motors are then deactivated to prevent crashes during robot movements. Sample loading starts with the pin and vial being picked from the HCD. On receiving a request to mount a sample, the system checks that there is no pin on the electromagnet nozzle and no vial in the Robot-Assistant. It also retracts the cryostream nozzle 20 mm from its original position. In case of an error, such as a pin or vial already present on the device, the system recovers using an automatic procedure before starting the mounting procedure (Fig. 4 ▸). Once the device is ready, three attempts are made to pick the sample before a failure is recorded. If sample picking is successful, an object-recognition camera then checks the sample holder as it exits the HCD. If the sample has been removed without a vial (usually owing to a vial being out of specification, weakness of the vial or pin magnetic strength, or ice blocking the vial in the puck) the RoboDiff drops the pin at a predefined location for later retrieval. If the sample holder is intact, it is brought to the beam position, where the vial is removed by the Robot-Assistant (Supplementary Movie S1). When picking the sample in the HCD, and when removing the vial by the Robot-Assistant, the six motor torques of the robot are measured to detect any mechanical clashes. If any are detected, the movement is automatically stopped and the sample holder is brought back to its initial position in the HCD, and a homing procedure of the goniometer axes is then automatically launched before moving to the next sample. As for sample picking, vial removal is attempted up to three times. If the vial cannot be removed, the sample is automatically returned to the HCD. During the entire movement, the vial remains vertical to retain LN_2_. Once the vial has been removed and is under the LN_2_ fountain at the Robot-Assistant park position, the cryostream nozzle is brought back to its original position to maintain the sample at 100 K by moving a pneumatic stage, and the robot arm is moved to χ = 0°. The trajectory of this latter movement ensures that the sample remains at the X-ray beam position and therefore in the centre of the flow of the cryostream. Data collection is currently performed in a standard orientation with the spindle axis horizontal (χ = 0°), but all other orientations from 0 to 90° are possible. However, the option of collecting at any χ angle ranging from 0 to 90° should not be confused with the functionality of κ goniometers (Brockhauser *et al.*, 2013 ▸) used to align unit-cell axes along the rotation axis of a goniometer. Nevertheless, the possibility of collecting data at χ ≠ 0° has several advantages, notably that the collection of data from the same crystal in different orientations in the X-ray beam may help to reduce systematic errors in an experiment (Weinert *et al.*, 2015 ▸). Additionally, when data for anomalous scattering experiments are collected at an absorption edge of an atom contained in the crystal, collecting data sets from the same crystal with the ω axis parallel (χ = 0°) and perpendicular (χ = 90°) to the direction of the polarization of synchrotron X-ray beams can provide additional phasing information (Schiltz & Bricogne, 2010 ▸).

Sample unloading follows the reverse procedure, but in this case the object-recognition cameras located on the third port of the HCD (Figs. 1 ▸ *a* and 3 ▸ *a*) are used to check for the absence of a vial at the unloading position. The empty vial held at the Robot-Assistant park position is filled with LN_2_, moved to the sample position and replaced on the pin. In case of icing, a stream of pressurized gas under the vial helps in this procedure. At the entrance of the HCD the presence of both the pin and vial is checked. If the vial is missing, the sample is dropped for later recovery. If not, the sample is replaced in its original position. The entire cycle of loading/unloading (see Supplementary Movie S1) takes an average of 50 s, not including optical loop centring and the movement of beamline components.

During loading, once the robot arm is at χ = 0°, the optical centre of rotation of the sample, as determined by the *MXCuBE* utility *LUCID* (https://github.com/mxcube/lucid), is brought to the beam position by moving the robot motors. Movements during data collection, including line and mesh scans, are then performed using the air-bearing spindle and the high-accuracy *YZ* translation stage beneath the robot (Fig. 1 ▸ *b*). For this, a MUSST synchronization card is used to gather the signals of the ω axis, vertical and horizontal encoder positions, the Pilatus3 2M detector command and state, the shutter command and state, the beam intensity before and after opening the fast shutter, and the motor position of the robot centring table (Nurizzo *et al.*, 2006 ▸). These data also serve as a diagnostic tool and link motor position and image number in order to find the best diffracting position of the crystal during X-ray centring protocols (Svensson *et al.*, 2015 ▸).

## 3. Results   

### 3.1. Test-data collections   

In order to demonstrate the data-collection capabilities of the RoboDiff, the results of three different data collections are presented in Table 2 ▸: the first to demonstrate data collection for a ‘standard’ molecular replacement (bovine trypsin), and the second (ferulic acid esterase, FAE) and third (thermolysin) to demonstrate the suitability of MASSIF-1 and RoboDiff for anomalous phasing experiments using diffraction data collected at wavelengths at or remote from the absorption edges of the anomalous scattering elements contained in crystals (Leonard *et al.*, 2005 ▸). SAD-optimized data-collection strategies [MXPressE SAD, a strategy optimized for high multiplicity (360°) with the resolution limit set to the value at which the *R*_merge_ between Bijvoet pairs is ≤5% with minimized radiation damage (Svensson *et al.*, 2015 ▸)] were used for FAE and thermolysin.

**Figure 6:**
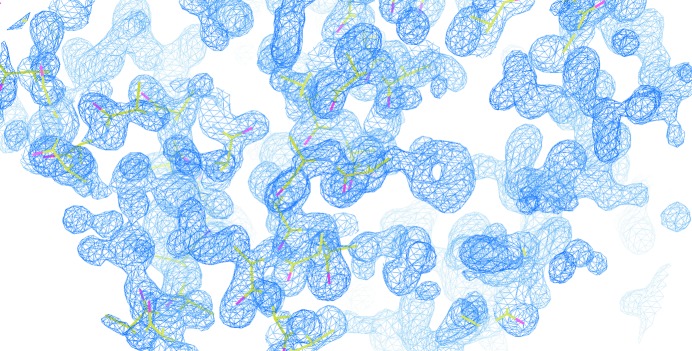
Experimentally phased map for thermolysin. A section of the experimentally phased map is shown contoured at 1.5σ with the C^α^ chain traced by *SHELXE*. The map demonstrates the quality of the phases calculated using data collected with the RoboDiff, as side chains and water molecules are clearly visible.

In all three cases diffraction images were indexed and integrated using *XDS* (Kabsch, 2010 ▸; Monaco *et al.*, 2013 ▸) and reduced using *AIMLESS* (Evans, 2011 ▸). For trypsin, the structure was solved by molecular replacement (MR) using *MOLREP* (Vagin & Teplyakov, 2010 ▸). To solve the structures of FAE and thermolysin, *SHELXD* was used to determine the ‘anomalous substructure’ (8 × Se and 5 × Cd^2+^ for FAE, 1 × Zn^2+^ for thermolysin) and *SHELXE* was used to derive phase probability distributions to *d*_min_ = 2.0 Å (Sheldrick, 2010 ▸). *ARP*/*wARP* (Perrakis, Morris *et al.*, 1999 ▸) was then run for automatic model building. Both models were completed manually using *Coot* (Emsley *et al.*, 2010 ▸) interspersed with refinement with *REFMAC*5 (Murshudov *et al.*, 2011 ▸). Tables 2 ▸ and 3 ▸ show the data-collection and refinement statistics from the three systems. The data-quality indicators and final electron-density maps (Fig. 6 ▸) for all crystals are excellent, demonstrating that the RoboDiff performs to the highest levels expected from a modern goniometer. In keeping with the ESRF data policy, the raw diffraction images for these data collections are available at Zenodo (http://zenodo.org/).

### 3.2. Performance   

The RoboDiff has been installed on the ESRF beamline MASSIF-1 since it opened to users in September 2014 and places no restrictions on the type (other than they must be SPINE compatible) or manufacturer of loops, pins or vials in which samples are mounted. Since then it has automatically handled over 20 000 samples from across the world from a wide range of biological projects. The device has been designed to maximize the use of beam time by escaping from potential problems and to continue mounting the available samples. In 2015, MASSIF-1 received 9872 samples, of which 272 could not be mounted or were lost, representing a global error rate of 2.7%. For the last 3440 sample-mount requests of 2015 detailed logs of all errors were recorded. This showed an error rate of 3.6% (125 errors recorded) but crucially, the type of error is known. This then breaks down evenly to 63 samples where the vial was lost on loading (1.8%) and 62 where the sample could not be mounted (see §[Sec sec2.5]2.5) and was replaced in the dewar (also 1.8%). The latter error is much less serious, as the sample is not lost and can often be mounted again. Occasionally, more serious errors occur that require manual intervention. The most serious is a robot crash, but the torque monitoring has reduced this occurrence to be insignificant.

Since receiving user samples, data collections have been performed on many types of crystal, from well characterized systems all the way to weakly diffracting crystals (Koromyslova *et al.*, 2015 ▸; Neudegger *et al.*, 2016 ▸; Papageorgiou *et al.*, 2016 ▸; Thierry *et al.*, 2016 ▸), with the smallest crystal having a volume of only 20 748 µm^3^ (28 × 57 × 13 µm; Bowler *et al.*, 2016 ▸), and 20 structures have been solved *de novo* to date (see, for example, Kharde *et al.*, 2015 ▸; Muir *et al.*, 2016 ▸). In combination with software routines that locate, centre and collect data (Svensson *et al.*, 2015 ▸), the RoboDiff has proved to not only be the cornerstone of complete automation but is also an instrument that can collect high-quality data from samples.

## 4. Conclusions   

The RoboDiff has been designed for high reliability and stability. This has been achieved by integrating a number of diagnostic tools in the sample changer and goniometer. Considerable effort has been made both to transfer the experience of beamline scientists to the software and to implement the ability to recover from situations that could otherwise prevent the continuation of experiments. To date, over 20 000 user samples have been loaded, characterized and collected. The 240 samples which can be stored in the HCD increase the throughput and allow MASSIF-1 to run unattended 24 h a day. The hardware and software is all owned by ESRF, making maintenance and developments very flexible. Moreover, several collaborations based on the RoboDiff project have been set up to allow dispersion of the technology all over the world. The close links that have been designed between the RoboDiff and the ISPyB database and *MXCuBE* brings MASSIF-1 to the forefront of instruments for structural biology, and provide one of the missing steps in the automatic gene-to-protein pipeline.

## Supplementary Material

PDB reference: trypsin, 5xfl

PDB reference: ferulic acid esterase, 5xfm

PDB reference: thermolysin, 5xfn

Supplementary Movie S1. Operation of the RoboDiff.. DOI: 10.1107/S205979831601158X/gm5047sup1.mp4

## Figures and Tables

**Table 1:** Error handling during sample transfer

	Error type	Detection	Origin	Solution
Load				
Before starting	Pin on magnet	Photo-interrupter	Mounted manually before/iced	Move to trash
	Vial on Robot-Assistant	Optical fibre	Left on Robot-Assistant/iced	Automatic blower
In the HCD	No pin on magnet	Photo-interrupter	No sample in puck	Next sample
			Non-SPINE pin	
Exit of the HCD	No vial on pin	Camera	Icing of the puck	Move to trash
			Vial out of specifications	Next sample
At the beam position	No vial on Robot-Assistant/vial cannot be removed	Optical fibre	Vial out of specifications	Replace sample in dewar
				Next sample
Unload				
At the beam position	No vial on Robot-Assistant	Optical fibre	Vial lost	Move to trash
				Next sample
	Vial cannot be placed on pin	Optical fibre	Icing	Automatic blower
				Wait 30 s, try again
Entrance of the HCD	No vial on pin	Camera	Icing or vial out of specifications	Move to trash
	No pin	Photo-interrupter		Next sample
In the HCD	Pin on magnet	Photo-interrupter	Pin out of specifications	Move to trash

**Table 2:** Data-collection strategy and statistics for trypsin, FAE and thermolysin Dose calculations were made using *RADDOSE*-3*D* (Zeldin *et al.*, 2013 ▸) using the crystal volume determined during the X-ray centring and the flux and beam profile (50 µm FWHM).

	Trypsin	FAE	Thermolysin
Crystal dimensions (µm)	531 × 83 × 89	286 × 108 × 102	700 × 43 × 178
Beam diameter (µm)	50	50	50
Wavelength (Å)	0.966	0.966	0.966
Unit-cell parameters (Å)	*a* = 61, *b* = 65, *c* = 70	*a* = *b* = 112, *c* = 66	*a* = *b* = 93, *c* = 130
Space group	*P*2_1_2_1_2_1_	*P*4_1_2_1_2	*P*6_1_22
Flux at sample position (photons s^−1^)	3.2 × 10^11^	3.6 × 10^11^	3.6 × 10^11^
Transmission (%)	100	100	100
Dose of the data collection (MGy)	4.58	16.03	13.15
Total exposure time (s)	96.5	295.5	251.8
Oscillation range (°)	33–160	0–360	0–360
Detector resolution (Å)	1.68	2.23	1.82
Anomalous signal† at 12.8 keV (%)	—	5.7	1.0
Mid-slope of anomalous normal probability	—	1.066	1.006
Figure of merit	—	0.65	0.45
Resolution range (Å)	47.5–1.78	49.9–1.99	46.6–1.45
Inner shell	47.5–6.91	49.9–7.41	46.6–5.62
Outer shell	1.85–1.78	1.98–1.99	1.50–1.45
SigAno‡	—	1.50	1.15
Inner shell	—	2.74	1.59
Outer shell	—	1.26	1.00
No. of unique reflections	26622	32088	55022
Inner shell	534	664	1215
Outer shell	2416	2640	3489
No. of observed reflections	123922	785881	1875128
Inner shell	2305	14912	39342
Outer shell	11374	59264	65613
Completeness (%)	98.7	98.3	92.5
Inner shell	99.2	99.7	99.8
Outer shell	93.1	83.8	61.4
Multiplicity	4.7	24.5	34.1
Inner shell	4.3	22.5	32.4
Outer shell	4.7	22.4	18.8
〈*I*/σ(*I*)〉	16.1	19.3	27.3
Inner shell	50.1	57.4	78.8
Outer shell	1.52	1.54	1.3
*R*_p.i.m._ § (%)	2.5	2.5	2.0
Inner shell	1.3	1.1	1.1
Outer shell	47.5	64.7	65.3
Wilson *B* factor (Å^2^)	30.6	31.01	19.84

† http://www.ruppweb.org/new_comp/anomalous_scattering.htm.

‡ SigAno is the mean anomalous difference in units of its estimated standard deviation [|*F*(+) − *F*(−)|/σ]. *F*(+) and *F*(−) are structure-factor estimates obtained from the merged intensity observations in each parity class.

§ *R*_p.i.m._ = 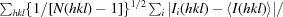 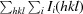, where 〈*I*(*hkl*)〉 is the average of symmetry-related observations of a unique reflection.

**Table 3:** Refinement statistics

	Trypsin	FAE	Thermolysin
PDB code	5fxl	5fxm	5fxn
Beamline	MASSIF-1	MASSIF-1	MASSIF-1
Space group	*P*2_1_2_1_2_1_	*P*4_1_2_1_2	*P*6_1_22
Wavelength (Å)	0.966	0.966	0.966
Phasing method	MR	SAD	SAD
Resolution range (Å)	20–1.78	20–1.91	20–1.45
*R* factor† (%)	18.2	19.0	14.4
Free *R* factor‡ (%)	21.0	19.7	18.0
*B* factors (Å^2^)			
Protein	18.6	24.7	19.4
Ligand	67.7	—	26.2
Water	41.0	36.8	31.3
R.m.s. deviations			
Bonds (Å)	0.872	0.721	0.681
Angles (°)	0.957	0.863	0.777

† *R* = 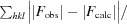 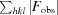, where *F*_obs_ and *F*_calc_ are the observed and calculated structure-factor amplitudes.

‡ *R*_free_ = 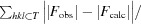 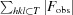, where *F*_obs_ and *F*_calc_ are the observed and calculated structure-factor amplitudes and *T* is the test set of data omitted from refinement (5% in this case).
